# The Role of Regulation and Emotional Eating Behaviour in the Early Development of Obesity

**DOI:** 10.3390/ijerph182211884

**Published:** 2021-11-12

**Authors:** Ana V. Valero-García, Marina Olmos-Soria, Julia Madrid-Garrido, Irene Martínez-Hernández, Emma Haycraft

**Affiliations:** 1 Department of Developmental and Educational Psychology, Faculty of Psychology, University of Murcia, 30100 Murcia, Spain; marolmos@um.es; 2 Pre-anaesthesia Unit, General University Hospital Santa Lucía, 30202 Cartagena, Spain; juliamadridgarrido@gmail.com; 3 Faculty of Education, International University of La Rioja (UNIR), 28040 Madrid, Spain; irene.martinez@unir.net; 4 School of Sport, Exercise and Health Sciences, Loughborough University, Loughborough LE11 3TU, UK; E.Haycraft@lboro.ac.uk

**Keywords:** eating behaviour, obesogenic behaviours, behavioural regulation, emotional self-regulation, emotional eating, parental regulation, childhood, obesity

## Abstract

The purpose of our research was to explore the role of both parents’ use of behavioural regulation with food and children’s emotional self-regulation in young children with and without overweight/obesity. For this purpose, 123 participants (*n* = 62 boys and *n* = 61 girls) were recruited and classified into two groups by their Body Mass Index (BMI, non-overweight vs. overweight/obese) and into two age groups (four years and seven years). The children’s parents/primary caregivers completed two scales of the Childhood Obesogenic Behaviours’ Questionnaire (COBQ). The participants were measured and weighed to calculate their BMI to identify overweight, obesity, and non-overweight. The results showed that the means for children who were obese/overweight were significantly higher than those of children who were non-overweight for both the parents’ behavioural regulation scale (non-overweight: *M* = 1.80, *SD* = 0.69; overweight/obesity: *M* = 2.94, *SD* = 0.85) and the child’s emotional overeating scale (non-overweight: *M* = 1.47, *SD* = 0.56; overweight/obesity: *M* = 2.65, *SD* = 0.87). No statistically significant differences were found related to age (4 and 7 years), indicating that the potential impact of obesogenic behaviours starts early in development. Similarly, no differences by gender were found. Due to the implications of obesity for physical and mental health, and the high probability of maintaining this overweight status in the long term, family-based interventions to prevent obesity are highly advisable from birth.

## 1. Introduction

Childhood obesity is a serious problem and is increasing in alarming proportions among infants, children, and adolescents [1]. The WHO [2] reported that 38 million children under the age of 5 were overweight or obese in 2019 and that 340 million children and adolescents aged 5–19 were overweight or obese in 2016. The implications of obesity in physical health (cardiovascular diseases, diabetes, etc.) are well known, but recently, research has also focused on its impact on mental health (self-esteem, depression, etc.). In the last decade, and related to the increase in child obesity mentioned above, its implications for development have been considered as it seems to affect several domains of personal and social adjustment [3], and has also been related to some mental health conditions (anxiety, low self-esteem, conduct problems, etc.) [4,5,6]. Nevertheless, most studies have been carried out in children over 8 years of age, so we still have little information on what influences obesogenic behaviours in earlier ages. A question to bear in mind is that the probability of suffering from obesity in later periods of life is extremely high in children who are obese/overweight. For example, 80% of adolescents with obesity have been estimated to maintain obesity in adulthood [7]. Additionally, even if the World Health Organization (WHO) suggests that we cannot talk properly about obesity but rather only a ‘risk of obesity’ for children under 5 years of age, the risk seems to be very serious, as a high percentage of children probably will develop obesity in childhood. In addition, considering that obesity is preventable, knowing the mechanisms underlying this rise in early obesity seems to be a priority.

Previous research has established links between eating behaviour and the early development of obesity. Eating behaviour is considered an interactive cycle, where a series of actions converge (including, for example, the influence of the family environment, food availability, genetic factors, etc.) to establish the child’s relationship with food in addition to the influence of social status, cultural traditions, and models of imitation and affective symbolisms [8,9,10]. Children develop their eating behaviour according to the strategies used by their parents [11] and through their direct experience with food. These early influences of feeding strategies by parents can have an impact on the development of obesogenic eating behaviour [12,13]. The importance of studying the home environment in the effective prevention and treatment of childhood obesity in order to better understand those factors linked to weight gain at an early age has been emphasised [14,15]. Thus, parents’ behavioural regulation has been associated with weight gain and higher caloric intake at an early age [16], as well as a child’s emotional overeating with increased caloric intake in stressful situations [17] and the development of greater obesogenic eating behaviour [18].

This role of emotional regulation on eating behaviour is consistent with several studies. For instance, a recent systematic review by Favieri, Marini, and Casagrande [19] found consistent negative relations, both in cross-sectional and longitudinal studies, between emotional regulation and overeating. A series of explanations have been offered for this relation, where factors related to parents and factors related to children should be considered. On the one hand, parents regulating their children’s behaviour by using food as a reward or punishment has been suggested. Thus, a mediational study [20] found that, when parents use food as a reward, their children’s ability to regulate their food intake decreases, which, in turn, is related to an increase in emotional overeating. On the other hand, some factors are related to the child themselves: for example, some authors have analysed their sensitivity to rewards as a possible mechanism underlying individual differences in children’s association between food and well-being [21]. However, factors related to temperamental traits have also been studied: children with higher negative emotionality are more prone to soothing themselves with food [22], and even low self-regulatory abilities at 2 years of age predicted being overweight at 5.5 years of age in a study by Graziano et al. [23]. In summary, though we do not know yet the deeper reasons, this relation between difficulties in emotional regulation and emotional eating seems clear in recent literature. Nevertheless, most studies have been carried out on adolescents and the ones conducted with children usually considered ages over seven. However, analysing younger ages is necessary if we want to clarify how these relations are established in order to prevent the early development of obesity and to understand its possible psychological and social implications on well-being. Some differences in gender have also been found in some studies, but again, these differences usually appear when samples of adolescents are considered [24,25].

The present cross-sectional study aims to understand more about obesogenic behaviours in young children 4 and 7 years of age, with attention focused on (1) the differential use of food by parents of children who are obese/overweight versus non-overweight as a way to regulate their children’s behaviour, and (2) studying eating as a means of emotional self-regulation in children who are obese/overweight compared with children who are non-overweight. Age and gender differences were also considered in both dimensions.

## 2. Materials and Methods

### 2.1. Participants

One hundred and twenty-three children, 62 girls and 61 boys aged four (*n* = 65) and seven (*n* = 58) years, participated in the study. Children within the appropriate age range with no physical or mental disability were recruited locally from nurseries and schools in Murcia, Spain. Following the guidelines from the Bioethical Institutional Review Board about data protection and privacy, consent forms were provided to parents and signed before participation in the study.

The study participants were distributed according to BMI classification (*N* = 69, obese/overweight; *N* = 54, non-overweight), age (*N* = 65, 4 years old; *N* = 58, 7 years old), and sex (*N* = 62, boys; *N* = 61, girls). The children in the study belonged to middle-class families and there were no differences between the broad socioeconomic level of the schools the children were recruited from.

### 2.2. Measures and Procedure

Following informed consent, children’s heights and weights were measured by a trained researcher at their nursery or school, and the parents were asked to complete a questionnaire on their children’s eating behaviours (see below). Their responses were returned in an envelope to their child’s nursery/school. Children’s heights and weights were measured to calculate BMI. The balance was the Vitalcontrol SBF 48 USB model (Vital Control, Balearic Islands, Spain), and the stadiometer was the portable Seca 213 (Seca Deutschland, Hamburg, Germany). For analysis purposes, we converted BMI to BMI Z-scores in accordance with the WHO Child Growth Standards reference data [26] adjusted for age and gender and into the following two groups according to their position on the WHO BMI distribution: ‘non-overweight’ (≥5th to ≤85th centile) and ‘overweight/obese’ (>85th to >95th centile) (Table 1).

The Childhood Obesogenic Behaviours’ Questionnaire (COBQ) [27]. The COBQ is a Spanish instrument that comprises 5 scales and 21 items. For the purpose of this study, only the Child Emotional Overeating and the Parent’s behavioural regulation scales were considered: (1) Child Emotional Overeating (4 items), which assesses if the child uses food in an emotional way (e.g., ‘When s/he is upset or has fought with someone, eating calms her/him down’); (2) Parent’s Behavioural Regulation (4 items), which measures parents’ use of food to regulate their children’s behaviours (e.g., ‘When my child behaves, I reward her/him with sweets, crisps, or another food to eat’. The parents were asked to rate the frequency of specific obesogenic-related behaviours on an ordinal scale, ranging from 1 (never) to 5 (always). The mean scores were calculated from the responses to each scale, and the possible scores ranged from one to five. Individual items were theoretically derived from research into the behavioural causes of obesity. The scales of the COBQ were found to display adequate internal validity and reliability in the current sample, and Cronbach’s alpha values were 0.91 (Parent’s Behavioural Regulation scale) and 0.94 (Child Emotional Overeating scale). Both scales were unidimensional, explaining at least 60% of the variance, and the two scales were correlated (0.901).

### 2.3. Data Analyses

To explore the differential use of food by parents of children who are obese/overweight as a way to regulate their children’s behaviour, a 2 × 2 × 2 factorial analysis of variance (ANOVA) was conducted, using the parent’s behavioural regulation scale of the COBQ as the dependent variable. The fixed factors were children’s BMI group (non-overweight and overweight/obesity), age (4 and 7 years), and gender (male or female). An effect size measuring the partial eta-squared was calculated, with values larger or equal to 0.06 but less than 0.14 being a medium effect size, and with values larger or equal to 0.14 being a large effect size [28].

To study eating as a means of emotional self-regulation, which was the second aim, a similar procedure was used with the scale for emotional overeating in a child as the dependent variable. The data were analysed using IBM SPSS Statistics for Windows (Version 22.0, Armonk, NY, USA).

## 3. Results

Differences by BMI Classification

**Figure 1:**
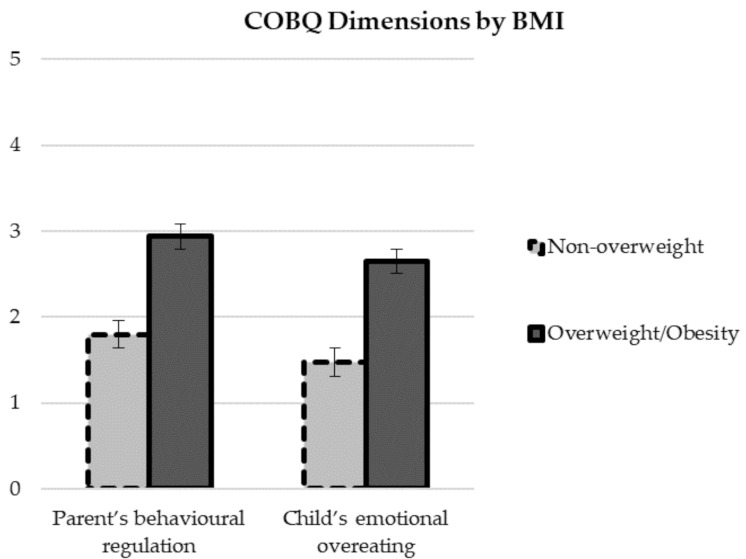
Differences in the Childhood Obesogenic Behaviours’ Questionnaire dimensions between children who are non-overweight and overweight/obese. No significant differences were found in the two scales considered of the Childhood Obesogenic Behaviours’ Questionnaire (Parent’s Behavioural Regulation and Child Emotional Overeating) between the two age groups and between boys and girls. Means for Parent’s Behavioural Regulation were 2.45 (*SD* = 0.99) for 4-year-olds and 2.41 (*SD* = 0.93) for 7-year-olds, and for Child Emotional Overeating means were 2.06 (0.96) for 4-year-olds and 2.22 (0.93) for 7-year-olds. As to gender, in the first variable means were 2.51 (0.90) for boys and 2.35 (1.02) for girls, and in Child Emotional Overeating means were 2.22 (0.95) for boys and 2.05 (0.95) for girls.

ANOVAs (BMI group x gender x age) for both COBQ scales were carried out. Residuals in the ANOVA model were not deviated from normality. The result for Levene test was F(7, 115) = 2.821; *p* < 0.01. Statistically significant effects were seen for the BMI group for both the parent’s behavioural regulation scale (F(1, 115) = 63.12; *p* < 0.001; *τ*^2^ = 0.354) and the child’s emotional overeating scale (F(1, 115) = 71.99; *p* < 0.001; *τ*^2^ = 0.385). The means for children who were obese/overweight were significantly higher than those of children who were non-overweight for both dimensions (Table 2 and Figure 1). None of the interactions between factors were statistically significant.

## 4. Discussion

The objective of this study was to explore the role of self-regulation and emotional overeating in the early development of obesity. To achieve our objective, we analysed two factors known to be involved in the relationship between emotional regulation and overeating: the first factor was the use of food by parents as a way to regulate their children’s behaviour, and the second factor was children’s emotional overeating. We considered differences between children who were obese/overweight and non-overweight at 4 and 7 years of age in a cross-sectional study.

Interestingly, the results indicate that parents of children who are overweight/obese report using food as a regulator to a greater extent than parents of non-overweight children. Thus, we conclude that the parents in this study whose children are obese/overweight are perhaps more likely to report rewarding appropriate behaviours by giving them food or punishing their children’s inappropriate behaviours by restricting the foods they liked best. Evidently, the interaction between parents and children, and the feeding styles that parents use to teach their children, is related to obesogenic eating behaviours [29]. On occasions, the power struggle between parents and children at mealtimes can generate emotional conflicts in children and can impact their eating behaviour [10], acting as a mediator between psychological problems and weight gain in children [5].

Our second objective was to know if children who are obese/overweight use emotional overeating from an early age, and the results show that this seems to be the trend: even 4-year-old children ask for food to soothe themselves when emotionally upset. Children who eat based on their emotions are more likely to develop obesogenic eating behaviours [18] and show a greater tendency towards the development of obesity [30]. These authors point out the difficulties that children who are obese or overweight can show to regulate their own emotions, resorting to large binges when faced with episodes of stress.

How one aspect relates to the other one is difficult to know: What is the direction of the influences? Are parents of children who are obese/overweight conditioning their children’s use of food, as some authors have pointed out [31,32]? Are children somewhat provoking their parents to use all available resources (food in this case) to calm down an irritable child, as some research on temperament suggests [22]? Do children have a special susceptibility to being easily conditioned (such as reward sensitivity) [21]? More questions remain than clear answers exist. Somehow, the intertwined relation between parent’s feeding styles and their children’s eating behaviour is complex.

Moreover, in this study, the lack of significant differences in Parent’s Behavioural Regulation and Child Emotional Overeating between the two age groups means that children who are obese/overweight are already behaviourally regulated by their parents, using food at four years of age and emotionally self-regulating with food. Some authors considered that children develop their behaviours and eating patterns in the first two years of life [33], while others stated that this development occurs during childhood [9]. Our results confirm that obesogenic eating behaviours are already learned at four years, in line with the former suggestion. In addition, this obesogenic pattern is the same at seven years of age. As mentioned above, we crossed the line between the age of potential risk of obesity and proper obesity. Genetic and environmental factors also have an impact on obesity, but the high probability of maintaining obesity mentioned above should be taken into account. Therefore, early age is a factor to consider when addressing family-based interventions to prevent the development of obesity.

The lack of significant differences in our study between boys and girls is congruent with previous literature at young ages. More differences between boys and girls in emotional regulation through food is mainly present in adolescence, probably due to the higher amount of worrying about good corporal images in girls [24].

The strengths of this study include the inclusion of two young age groups and the comparison of obese/overweight and non-overweight groups of children. In future research, the use of a longitudinal design to follow the development of risks for obesity and to consider factors influencing younger ages is advisable. Expanding the sample and focusing on aspects, such as the emotional self-regulation of parents to observe their impact on the learned behaviours of children with obesity, would also be interesting.

## 5. Conclusions

In conclusion, parents of children who are obese/overweight use food to regulate their children’s behaviour and children who are obese/overweight emotionally self-regulate with food. This pattern has been found for children as young as 4 years of age and for 7-year-old children. Thus, a relationship between family behaviours and children’s weight status is clear from as early as 4 years of age. As this relationship probably starts earlier in development, and perhaps from birth, targeting family-based obesity interventions at very early ages is advisable.

## Figures and Tables

**Table 1:** Characteristics of the sample by age.

	Age		
Gender/BMI z-Scores	4 Years	7 Years	Total
Children who are non-overweight	30	24	54
Boys who are non-overweight	17	9	26
Girls who are non-overweight	13	15	28
Children who are overweight/obese	35	34	69
Boys who are overweight/obese	18	18	36
Girls who are overweight/obese	17	16	33
Total	65	58	123

**Table 2:** Measures for the two scales of the Childhood Obesogenic Behaviours’ Questionnaire by BMI classification.

COBQ Measures	Non-Overweight	Overweight/Obesity	*p*
Parent’s behavioural regulation	1.80 (0.69)	2.94 (0.85)	<0.001
Child’s emotional overeating	1.47 (0.56)	2.65 (0.87)	<0.001

## Data Availability

The data presented in this study are available on request from the corresponding author.
